# Inhibition of HDAC6 promotes microvascular endothelial cells to phagocytize myelin debris and reduces inflammatory response to accelerate the repair of spinal cord injury

**DOI:** 10.1111/cns.14439

**Published:** 2023-08-29

**Authors:** Chengjie Wu, Yalan Pan, Lining Wang, Mengmin Liu, Pengcheng Tu, Sixian Chen, Lei Shi, Danqing Yan, Yong Ma, Yang Guo

**Affiliations:** ^1^ Department of Traumatology and Orthopedics Affiliated Hospital of Nanjing University of Chinese Medicine Nanjing China; ^2^ Laboratory of New Techniques of Restoration & Reconstruction, Institute of Traumatology & Orthopedics Nanjing University of Chinese Medicine Nanjing China; ^3^ Laboratory of Chinese Medicine Nursing Intervention for Chronic Diseases Nanjing University of Chinese Medicine Nanjing China; ^4^ School of Chinese Medicine, School of Integrated Chinese and Western Medicine Nanjing University of Chinese Medicine Nanjing China

**Keywords:** autophagy‐lysosome, HDAC6, immune regulation, inflammatory response, microvascular endothelial cells, myelin debris, spinal cord injury

## Abstract

**Aims:**

To identify an effective strategy for promoting microvascular endothelial cells (MECs) to phagocytize myelin debris and reduce secretion of inflammatory factors following spinal cord injury (SCI).

**Methods:**

We established a coculture model of myelin debris and vascular‐like structures. The efficiency with which MECs phagocytize myelin debris under different conditions was examined via ELISA, flow cytometry, and immunofluorescence. Tubastatin‐A was used to interfere with the coculture model. The anti‐inflammatory effects of Tubastatin‐A were observed by HE staining, flow cytometry, immunofluorescence, and ELISA.

**Results:**

MECs phagocytized myelin debris via IgM opsonization, and phagocytosis promoted the secretion of inflammatory factors, whereas IgG‐opsonized myelin debris had no effect on inflammatory factors. Application of the HDAC6 inhibitor Tubastatin‐A increased the IgG levels and decreased the IgM levels by regulating the proliferation and differentiation of B cells. Tubastatin‐A exerted a regulatory effect on the HDAC6‐mediated autophagy‐lysosome pathway, promoting MECs to phagocytize myelin debris, reducing the secretion of inflammatory factors, and accelerating the repair of SCI.

**Conclusions:**

Inhibition of HDAC6 to regulate the immune‐inflammatory response and promote MECs to phagocytize myelin debris may represent a novel strategy in the treatment of SCI.

## Graphical abstract

Tubastatin‐A promotes MECs to phagocytize myelin debris by regulating the immune‐inflammatory response. Inhibition of HDAC6 after SCI regulates B cell proliferation and differentiation, upregulates IgG, downregulates IgM, and reduces inflammatory factors secreted by MECs following the phagocytosis of myelin debris. In contrast, inhibition of HDAC6 regulates the autophagy‐lysosome pathway to promote MECs to phagocytize myelin debris and reduce the secretion of inflammatory factors.

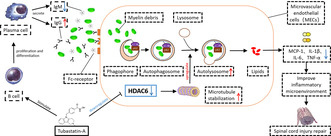

## 1 INTRODUCTION

Spinal cord injury (SCI) has been associated with high rates of disability, substantially impacting the quality of life of affected patients while increasing social and economic burdens. Primary traumatic injury leads to neuronal apoptosis and axonal rupture. Early control of secondary injury is key to improving treatment outcomes in patients with SCI.^1^

Following SCI, the myelin sheath is severely damaged, and the inflammatory microenvironment associated with the presence of large amounts of myelin debris can substantially hinder SCI repair. Furthermore, microglia and astrocytes, which are responsible for phagocytizing myelin debris, are absent in the early stages of SCI.^2^, ^3^, ^4^, ^5^, ^6^ Zhou et al.^7^ recently reported that microvascular endothelial cells (MECs) can phagocytize myelin debris via opsonization of immunoglobulin G (IgG)—a process dependent on the autophagy‐lysosome pathway, which plays an important role in improving the inflammatory microenvironment in the early stages of SCI. However, phagocytizing myelin debris can stimulate MECs to secrete inflammatory factors, which may in turn aggravate secondary injury.^7^, ^8^ Therefore, strategies that promote MECs to phagocytize myelin debris without inducing the secretion of inflammatory factors have become a critical area in SCI research.

The autophagy‐lysosome pathway is a self‐degradation process that removes misfolded or aggregated proteins, damaged organelles, and unnecessary or dysfunctional cellular components. This regulatory mechanism is necessary for maintaining axonal homeostasis in the nervous system.^9^, ^10^ Majora et al.^11^ observed that enhancing the transport function of the microtubule system can promote the fusion of autophagosomes and lysosomes, thereby increasing autophagic flux. Furthermore, Zheng et al.^12^ confirmed that the degree of microtubule stabilization can affect the autophagy‐lysosome pathway. Based on these findings, researchers have suggested that microtubule system stabilization is closely related to the autophagy‐lysosome pathway and may represent an important regulatory pathway for the phagocytosis of myelin debris by MECs.

Posttranslational acetylation of tubulin plays an important role in regulating the structure and stability of microtubules and has been shown to affect intracellular signal transduction, cell migration, and neuropathy.^13^ The histone deacetylase (HDAC) family contains many deacetylases that differ in structure and substrate specificity, enzymatic mechanisms, and subcellular localization. Some studies have shown that HDAC6 regulates the stabilization of the cellular microtubule system, intracellular material transport, and migration. A close association between HDAC6 and the development of various nervous system diseases has also been reported.^14^, ^15^ Following SCI, significant increases in HDAC6 expression can alter microtubule stabilization and lead to disorders of autophagy‐lysosome transport along the microtubule system.^16^ Notably, research has demonstrated that specific inhibition of HDAC6 can significantly inhibit the expression of interleukin (IL)‐1β and IL‐6, alleviate the local inflammatory response, and reduce neuronal apoptosis.^17^, ^18^

In this study, we established a coculture model of myelin debris and vascular‐like structures to aid in identifying an effective strategy for promoting MECs to phagocytize myelin debris while attenuating the secretion of inflammatory factors.

## 2 METHODS

### 2.1 Animals

Female C57BL/6 mice (6–8 weeks old) purchased from the Qinglong Mountain Animal Breeding Farm in Nanjing (license number SYXK [Su] 2019‐0010) were used for all experiments. Mice were reared under specific pathogen‐free conditions and were allowed free access to food and water at any time. All animal procedures were approved by the Animal Ethics Committee of Nanjing University of Chinese Medicine (201912A023) and were carried out in accordance with the guidelines of the Ministry of Public Health of China on the Care and Use of Laboratory Animals and the ARLET guidelines (Animal Research: Reporting in vivo experiments).

### 2.2 Cells

Mouse brain microvascular endothelial cells (BEND3) and mouse monocytic macrophage leukemia cells (RAW264.7) were purchased from Procell Life Science & Technology Co., Ltd. (CL‐0598, CL‐0190) and were cultured in DMEM under high‐glucose conditions (containing 10% FBS and 1% P/S).

### 2.3 Induction of SCI model

The SCI model was established in C57BL/6 mice using the modified Allen method.^19^ Briefly, mice were anesthetized via an intraperitoneal injection of 1% pentobarbital sodium (80 mg/kg). After iodophor disinfection, the skin was cut longitudinally along the spine with a scalpel, the T9‐T11 lamina was exposed, the T10 lamina was opened and cutoff, and a spinal cord percussion device was used to hit the T10 spinal cord. Immediate elimination of tension in the hind limbs indicates the successful establishment of the model. After surgery, the mice were placed on a cotton pad until they were fully awake. Their bladders were emptied manually daily to prevent urinary tract infections until the mice were able to urinate independently. Mice in the sham group underwent the same procedures without any impact on the spinal cord.

### 2.4 Myelin debris in the mouse brain

C57BL/6 mice were euthanized under anesthesia. The brain tissue was removed with tweezers and placed in a 0.32 M precooled sucrose solution, cut into small pieces, repeatedly broken into homogenates, and diluted with 0.32 M sucrose solution. An equal amount of 0.85 M sucrose solution was slowly added. After centrifugation for 45 min at 25,000 rpm, crude myelin debris was collected between the two sucrose solutions. After several rounds of grinding and centrifugation, the supernatant was discarded. Finally, after weighing, the myelin debris was packed in 1.5 mL centrifuge tubes at 100 mg/mL and stored at −80°C.^7^

### 2.5 Coculture system including MECs and myelin debris

The cultured MECs were inoculated on climbing slices of 12‐well plates (coated with matrix glue) at a density of 1 × 10^5^ per well, forming a vascular‐like structure after 24 h of culture. The prepared myelin debris was labeled with 50 μM CFSE, mixed, and incubated at 37°C for 15 min. Aseptic PBS containing 100 mM glycine was added, and the cells were resuspended. After incubation for 5 min at 37°C, the myelin debris was centrifuged at 12,000 rpm for 10 min, resuspended in aseptic PBS, centrifuged, and remixed, and finally resuspended to 100 mg/mL. Myelin debris (1 mg/mL) was added to the vascular‐like structure to form a coculture containing MECs and myelin debris.

### 2.6 Drug intervention

Following the SCI procedure, mice in the Tubastatin‐A group were intraperitoneally injected with Tubastatin‐A (Yuanye, S80226) at 50 mg/kg/day,^20^ while mice in the model and sham groups were injected with the same amount of saline solution. For the cellular experiment, MECs from the Tubastatin‐A group were treated with 40 μM Tubastatin‐A, while MECs of the Baf‐A1 group were treated with 50 nM bafilomycin A1 (Abcam, ab120497).^16^ These indices were measured after 72 h.

### 2.7 Transwell cultures

MECs and myelin debris were cocultured for 72 h, washed three times with PBS, and cultured for 24 h. The supernatant was collected and centrifuged at 12,000 rpm for 10 min, and the resulting supernatant was again collected. For the coculture experiments, the supernatant was added to the lower chamber of an 8‐μm transwell plate, while mononuclear macrophages were added to the upper chamber. After culturing for 6 h, the diaphragm was removed, stained with a crystal violet staining kit (Beyotime, C0121), and observed under a microscope.

### 2.8 Hematoxylin and eosin (HE) staining and Nissl staining

Paraffin sections (3–4 μm) of tissue surrounding the lesion site in the spinal cord were stained with H&E (Servicebio, G1005) and Nissl (Servicebio, G1036) staining kits. After adding neutral gum to the slice, the slide was covered, and the sections were observed under a microscope.

### 2.9 Transmission electron microscopy

After discarding the culture medium, an appropriate amount of electron microscope fixation solution (Servicebio, G1102) was added without rinsing. A cell scraper was used to scrape the cells gently, which were cut into ultrathin sections (60–80 nm) after centrifugation, fixation, rinsing, dehydration, embedding, and infiltration. The sections were dried overnight at room temperature after double staining with uranium and lead and later observed under a transmission electron microscope (Hitachi, SU8100).

### 2.10 Enzyme‐linked immunosorbent assays (ELISA)

The supernatant and serum were collected and analyzed using a mouse IL‐1β ELISA kit (mlbio, ml301814), IL‐6 ELISA kit (mlbio, ml063160), IL‐10 ELISA kit (mlbio, ml037873), MCP‐1 ELISA kit (mlbio, ml037840), IgG ELISA kit (mlbio, ml037601), IgM ELISA kit (mlbio, ml063597), TNF‐α ELISA kit (mlbio, ml002095), C3 ELISA kit (mlbio, ml002033), and MBP ELISA kit (Jin Yibai, JEB‐12718). The absorbance of each well was measured at 450 nm.

### 2.11 Immunofluorescence staining

For cell climbing experiments, the slices were washed three times with PBS, fixed with 4% paraformaldehyde for 10 min, and permeabilized with 0.2% Triton‐100 for 10 min. The slices were then washed three times with PBS and sealed with 5% goat serum for 30 min at room temperature. For paraffin sections, antigen repair was performed after dewaxing, and the sections were sealed with 5% BSA at room temperature. The primary antibodies used in this study were obtained from the following sources and were used at the following dilutions: CD31 (1:500, Affinity, AF6191), HDAC6 (1:500, Proteintech, 12,834‐1‐AP), MBP (1:500, Proteintech, 15,089‐1‐AP), LC3B (1:500, Proteintech, 18,725‐1‐AP), NLRP3 (1:500, Bioss, bs‐6655R), CD11b (1:500, Bioss, bs‐1014R), and GFAP (1:500, CST, 3670). After overnight incubation at 4°C, the sections were again washed three times with PBS. The secondary antibodies used in this study were as follows: Alexa‐488 (1:500, Abcam, ab150077), Alexa‐594 (1:500, Abcam, ab150080), FITC (1:500, Servicebio, GB22303), Cy3 (1:500, Servicebio, GB21303), and Cy5 (1:500, Servicebio, GB273030). After incubation at room temperature for 1 h, sections were again washed three times with PBS. Fluorescent multiple staining was performed using a TSA Plus kit (Servicebio, G1236). After re‐staining with DAPI, sections were washed with PBS, and a fluorescence inverted microscope or laser confocal microscope was used to observe and obtain images after adding an anti‐fluorescence quenching agent.

### 2.12 Flow cytometry and magnetic bead separation

MEC preparations were washed three times in PBS. After digestion, the cells were centrifuged at 1000 rpm for 5 min. After washing, centrifugation, and resuspension, the samples were subjected to flow cytometry. For B cells, C57BL/6 mice were sacrificed under excessive anesthesia, and the spleen was removed and placed on a 200‐mesh cell filter. The spleen was ground with a syringe, and RPMI‐1640 medium was added simultaneously. After collecting the filtrate, lymphocytes were separated using a lymphocyte separation kit (Solarbio, P8860) and centrifuged at 1000 rpm for 10 min. Then, the supernatant was discarded, and the cells were resuspended. Ten microliters of CD19 magnetic beads (Miltenyi, 130‐121301) were added, and the preparation was incubated at 4°C for 10 min. Magnetic beads were separated using a sorting column and magnetic equipment. After centrifugation, the B cells were cultured in an incubator. They were collected and centrifuged 24 h later, after which the supernatant was discarded, and the cells were resuspended. After adding 10 μL of Fc receptor blocker (Miltenyi, 130‐09257575), the cells were incubated at 4°C for 10 min. Then, CD19‐APC (BD, 550992) and CD138‐PE (Biolegend, 142504) were added at a volume of 1 μL each, and the cells were incubated at 4°C for 30 min in the dark. After washing, centrifugation, and resuspension, the samples were finally tested using flow cytometry.

### 2.13 Western blotting and co‐immunoprecipitation (co‐IP) experiments

RIPA lysate (containing 1% phosphatase and protease inhibitor) was added to the cell plate, and the cells were dissociated on ice for 30 min and centrifuged at 13,000 rpm for 10 min at 4°C. After the supernatant was collected, the protein content was detected using the BCA method. Upon addition of the loading buffer, the supernatant was placed in a boiling water bath for 10 min. A precast gel (ACE, F15420Gel) was used for electrophoresis. The proteins were transferred to an activated PVDF membrane, which was sealed with 5% skimmed milk for 2 h at room temperature and rinsed with TBST. The primary antibodies used in this study were as follows: HDAC6 (1:1000, Proteintech, 12,834‐1‐AP), Acetyl‐α‐Tubulin (1:1000, CST, 5335S), α‐Tubulin (1:5000, Proteintech, 11,224‐1‐AP), Kinesin‐5 (1:5000, Abcam, ab167429), MBP (1:1000, Proteintech, 15,089‐1‐AP), LC3B (1:1000, Proteintech, 18,725–1AP), p62 (1:1000, Proteintech, 18,420‐1‐AP), p‐ULK1 (1:2000, Proteintech, 80,218‐1‐RR), ULK1 (1:1000, Proteintech, 20,986‐1‐AP), Beclin1 (1:1000, Proteintech, 11,306‐1‐AP), IL‐1β (1:1000, Affinity, AF5103), IL‐6 (1:1000, Affinity, DF6087), TNF‐α (1:5000, Proteintech, 60,291‐1‐Ig), GAPDH (1:10000, Proteintech, 60,004‐1‐Ig), β‐actin (1:10000, Proteintech, 66,009‐1‐Ig), Syntaxin17 (1:1000, Proteintech, 17,815‐1‐AP), and VAMP8 (1:1000, Proteintech, 15,546‐1‐AP). After incubation overnight at 4°C, membranes were washed three times with TBST. The secondary antibodies used in this study were as follows: goat anti‐rabbit HRP (1:5000, Affinity, S0001) and goat anti‐mouse HRP (1:5000, Affinity, S0002). After incubation at room temperature for 1.5 h, membranes were washed three times in TBST. ECL was added prior to analysis using a gel imaging system. For Co‐IP experiments, the rProtein A/G Magnetic IP/Co‐IP Kit (ACE, AM001‐01) was used for detection, and all other steps were the same as those used for western blotting.

### 2.14 Statistical analysis

Two‐tailed unpaired Student's *t* test was used to determine the significance of differences between the two groups. Differences among multiple groups were analyzed using one‐ or two‐way analyses of variance (ANOVA), followed by Tukey's post hoc test. Normality was assessed using the Shapiro–Wilk test, and the data with nonnormal distribution were evaluated using the Mann–Whitney *U* test. All statistical analyses were performed using GraphPad Prism version 8. Values are expressed as mean ± standard deviation (SD). Statistical significance was set at *p* < 0.05.

## 3 RESULTS

### 3.1 Phagocytosis of myelin debris by MECs triggers an inflammatory response

**FIGURE 1:**
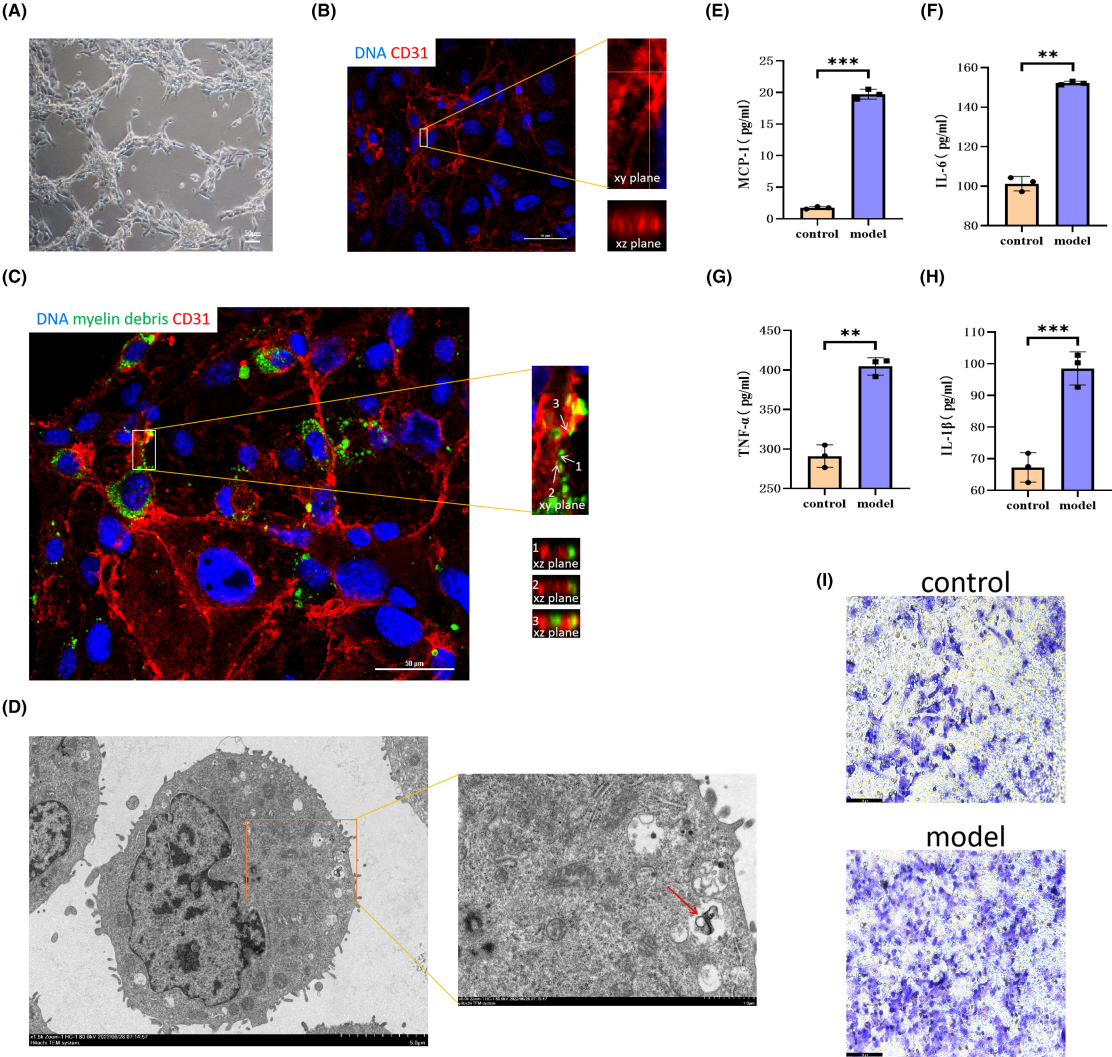
Phagocytosis of myelin debris by microvascular endothelial cells (MECs) increases the secretion of inflammatory factors. (A, B) MECs were inoculated on the matrix glue, and the x‐y and x‐z views show the formation of the tubular structure, indicating the successful generation of a vascular‐like structure (CD31, red). (C) After 72 h of coculture, we examined the dynamic process of myelin debris entering vascular‐like structures using x‐y and x‐z views (myelin debris, green): 1. The myelin debris was close to the vascular‐like structure. 2. The myelin debris had just come into contact with the vascular‐like structure. 3. The myelin debris had completely entered the vascular‐like structure. (D) The microstructure of MECs was observed using a transmission electron microscope. The red arrow indicates endocytosis of myelin debris. (E‐H) Levels of IL‐1β, IL‐6, MCP‐1, and TNF‐α in the supernatant were examined via ELISA (myelin debris was cocultured with MECs in the model group). (I) A transwell experiment was performed to examine the effect of phagocytosis of myelin debris by MECs on macrophage recruitment (macrophages through micropores were stained purple). All data are expressed as the mean ± standard deviation (*n* ≥ 3 replicates per group). **p < 0.01, ***p < 0.001.

We first constructed a co‐culture system of MECs and myelin debris, confirming that MECs phagocytized myelin debris based on the results of 3D confocal microscopy and transmission electron microscopy analyses (Figure 1A–D). Next, ELISAs of the supernatant revealed that levels of IL‐1β, IL‐6, MCP‐1, and TNF‐α were upregulated after phagocytosis of myelin debris by MECs (Figure 1E–H). Last, results from the Transwell experiment verified that phagocytosis of myelin debris by MECs induced macrophage recruitment (Figure 1I). The above experimental results were consistent with those of Zhou et al.,^7^ suggesting that phagocytosis of myelin debris by MECs aggravates the inflammatory response.

### 3.2 Phagocytosis of myelin debris by MECs requires opsonization of IgM or IgG, while IgM aggravates the inflammatory response

**FIGURE 2:**
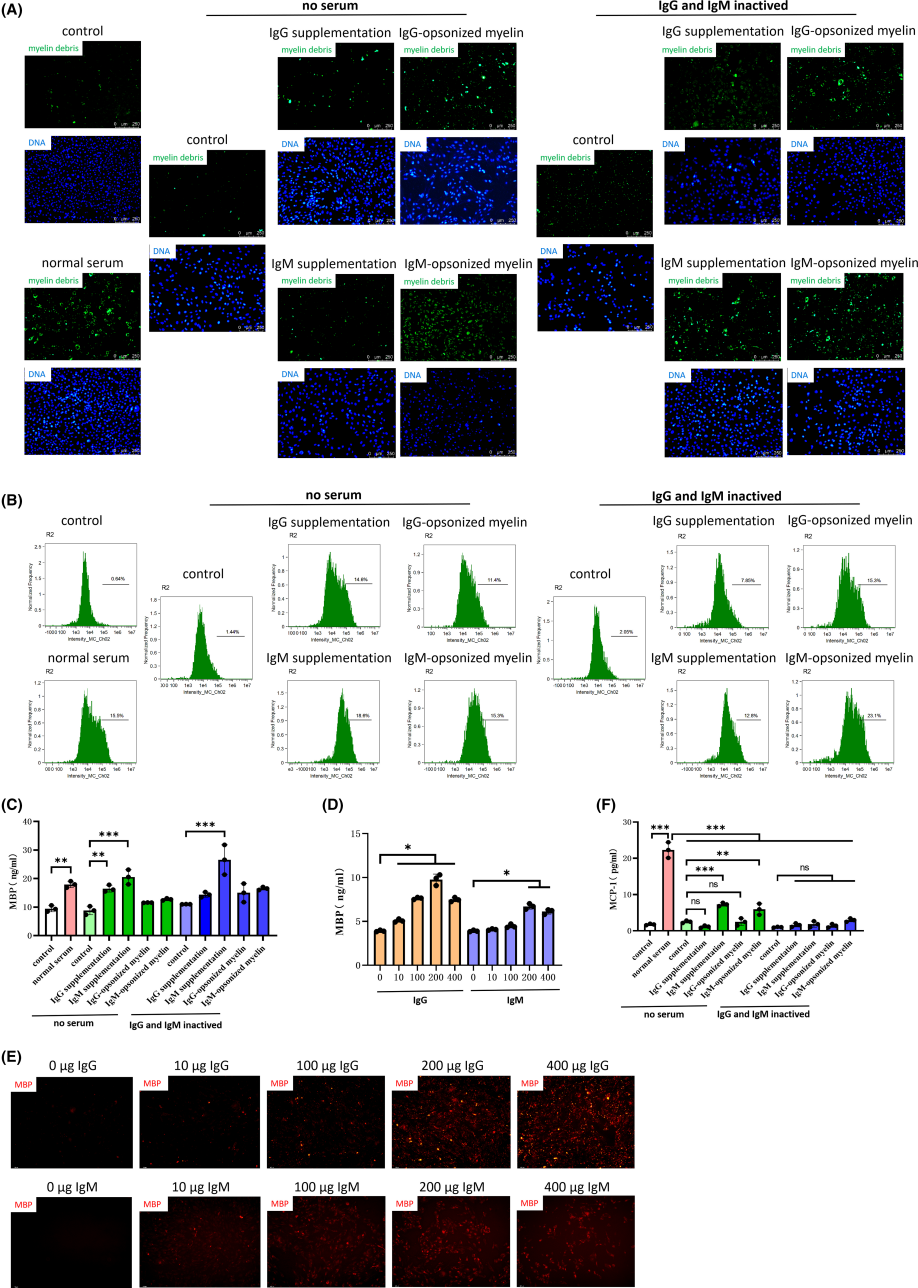
Opsonization of IgM or IgG affects the efficiency of phagocytosis. (A–C) The efficiency with which microvascular endothelial cells (MECs) phagocytize myelin debris under different conditions was examined via ELISA, flow cytometry, and immunofluorescence (myelin debris, green). In the absence of serum or in case of inactivation of IgM and IgG, supplementation with IgM, IgG, or myelin debris opsonized by IgM or IgG increased the efficiency of phagocytosis. (D, E) IgM and IgG were added at concentrations of 0 μg/mL, 10 μg/mL, 100 μg/mL, 200 μg/mL, and 400 μg/mL to interfere with the coculture system (myelin debris, red). Immunofluorescence and ELISA experiments revealed a positive correlation between the concentration of IgM or IgG and the efficiency with which MECs phagocytized myelin debris. (F) After collecting the cell supernatant, ELISA experiments were performed without serum and with inactivation of IgG; supplementation of IgG or myelin debris opsonized by IgG had no effect on MCP‐1 levels. However, when experiments were performed without serum, supplementation of IgM or myelin debris opsonized by IgM upregulated MCP‐1 levels. All data are expressed as the mean ± standard deviation (*n* ≥ 3 replicates per group). ns *p* > 0.05, *p < 0.05, **p < 0.01, ***p < 0.001.

To explore the specific mechanism by which phagocytosis of myelin debris by MECs leads to the secretion of inflammatory factors, we designed a serum‐free group and an IgG inactivation group by heating the serum to 70°C. Immunofluorescence and flow cytometry experiments indicated that phagocytosis of myelin debris by MECs depended on the opsonization of IgG (Figure 2A,B), similar to the results reported by Zhou et al.^7^ Some studies have suggested that IgM is the primary immunoglobulin produced by the primary immune response, which exhibits high opsonization efficiency.^21^ Therefore, we also performed immunofluorescence, flow cytometry, and ELISA experiments in an IgM group, which indicated that phagocytosis of myelin debris by MECs also required the opsonization of IgM (Figure 2C). We then explored the effects of different concentrations of IgG and IgM on the phagocytosis of myelin debris by MECs. Immunofluorescence and ELISA results revealed that the efficiency with which MECs phagocytized myelin debris depended on IgG or IgM (Figure 2D,E). Considering that no previous studies have reported the secretion of inflammatory factors after phagocytosis of myelin debris opsonized by IgM, we examined the supernatant from each group using ELISA. The results indicated that phagocytosis of myelin debris opsonized by IgM was associated with an increase in the level of MCP‐1, whereas no such effect was observed for IgG opsonization (Figure 2F).

### 3.3 Inhibition of HDAC6 promotes MECs to phagocytize myelin debris and reduces the inflammatory response

**FIGURE 3:**
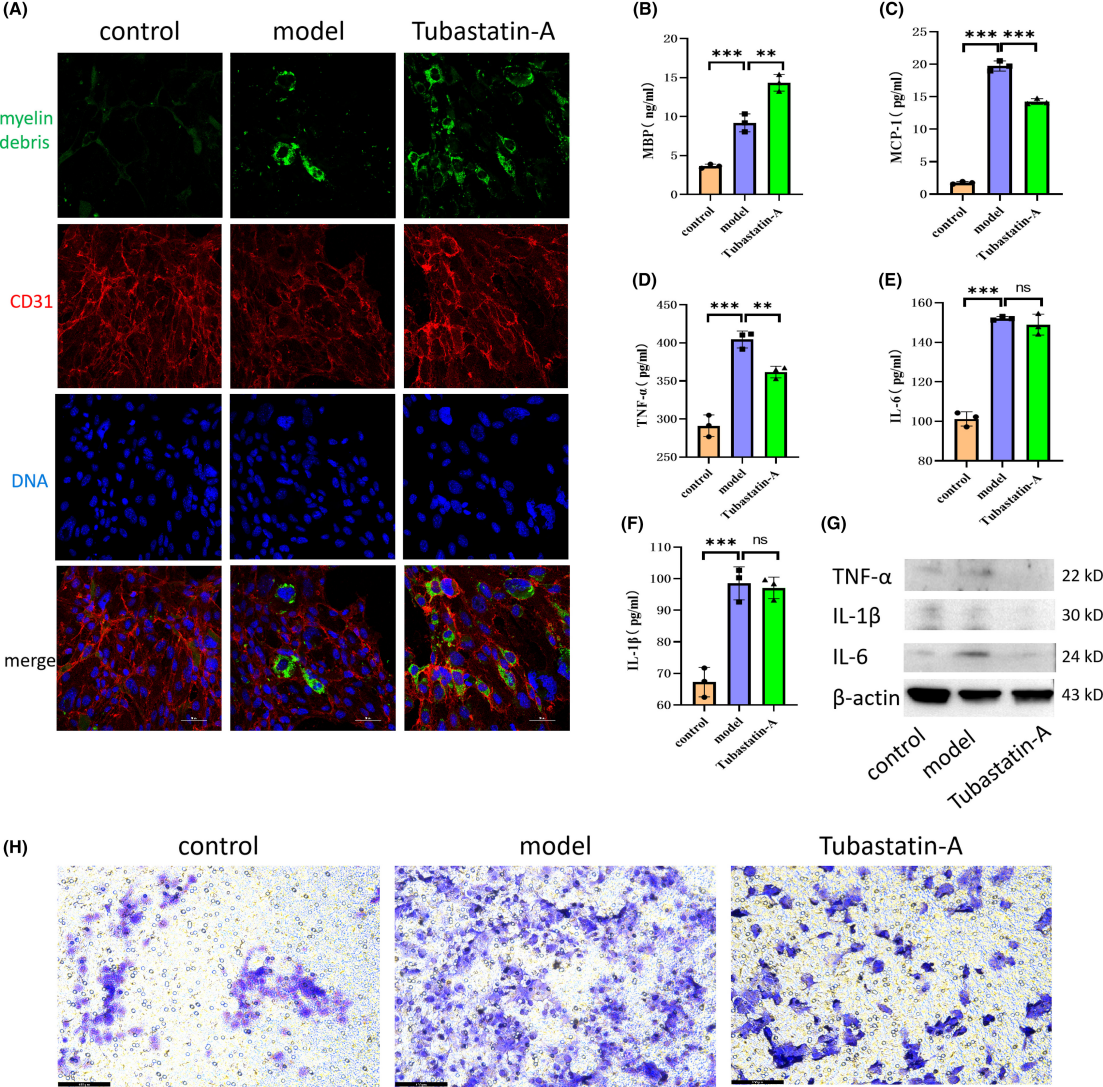
Tubastatin‐A increases the efficiency with which microvascular endothelial cells (MECs) phagocytize myelin debris and decrease inflammatory secretion. (A, B) Tubastatin‐A was used to interfere with the coculture system (myelin debris, green). Immunofluorescence and ELISA experiments revealed that Tubastatin‐A increased the efficiency with which MECs phagocytize myelin debris and upregulated intracellular MBP levels. (C–G) Tubastatin‐A was used to interfere with the coculture system. Western blot and ELISA experiments revealed that Tubastatin‐A reduced IL‐1 β, IL‐6, MCP‐1, and TNF‐α content in the cell supernatant. (H) The effect of phagocytosis of myelin debris by MECs on macrophage recruitment (macrophages passed through micropores were stained purple) was examined in a transwell experiment. All data are expressed as the mean ± standard deviation (*n* ≥ 3 replicates per group). ns *p* > 0.05, **p < 0.01, ***p < 0.001.

Some studies have suggested that Tubastatin‐A (an HDAC6 inhibitor) can reduce the inflammatory response after SCI.^17^, ^18^ Thus, we used Tubastatin‐A to interfere with the coculture system, observing that it promoted MECs to phagocytize myelin debris while reducing levels of IL‐1β, IL‐6, MCP‐1, and TNF‐α (Figure 3A–G). The Transwell experiment also indicated that Tubastatin‐A reduced macrophage recruitment caused by myelin debris (Figure 3H). These results suggest that inhibition of HDAC6 promotes MECs to phagocytize myelin debris and attenuates the inflammatory response, representing a novel potential treatment strategy for SCI.

### 3.4 HDAC6 inhibition increases IgG levels and decreases IgM levels by regulating the differentiation of B cells

**FIGURE 4:**
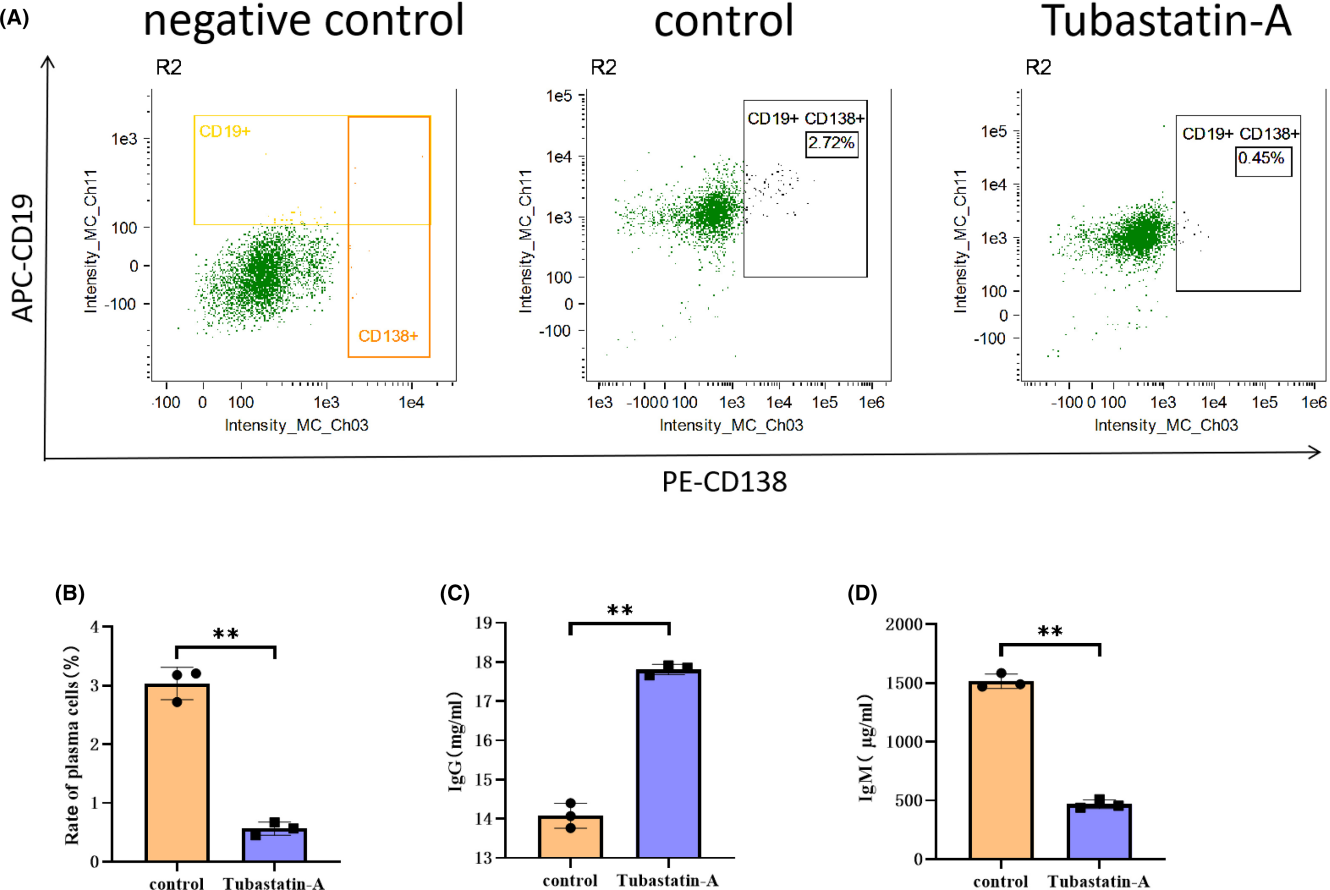
Tubastatin‐A increases IgG levels and decreases IgM levels by regulating the differentiation of B cells. (A, B) Tubastatin‐A was used to interfere with primary B cells obtained from mice using CD19 magnetic beads. Flow cytometry experiments revealed that Tubastatin‐A inhibited the differentiation of B cells into plasma cells. (C, D) ELISA of the cell supernatant revealed that Tubastatin‐A increased IgG levels and decreased IgM levels. All data are expressed as the mean ± standard deviation (*n* ≥ 3 replicates per group). **p < 0.01.

In the early stages of SCI, the accumulation of myelin debris at the local injury site induces an immune response. IgM and IgG produced by plasma cells (effector B cells) play a role in opsonization,^22^ and the regulation of HDAC6 may affect the differentiation of B cells; however, flow cytometry results indicated that Tubastatin‐A reduced this differentiation (Figure 4A,B). ELISA experiments further revealed that Tubastatin‐A increased IgG levels while decreasing IgM levels (Figure 4C,D). These results suggest that inhibition of HDAC6 regulates B cell differentiation, increases IgG levels, and decreases IgM levels to downregulate the level of MCP‐1, helping to attenuate the inflammatory response.

### 3.5 Inhibition of HDAC6 promotes MECs to phagocytize myelin debris by regulating the autophagy‐lysosome pathway

**FIGURE 5:**
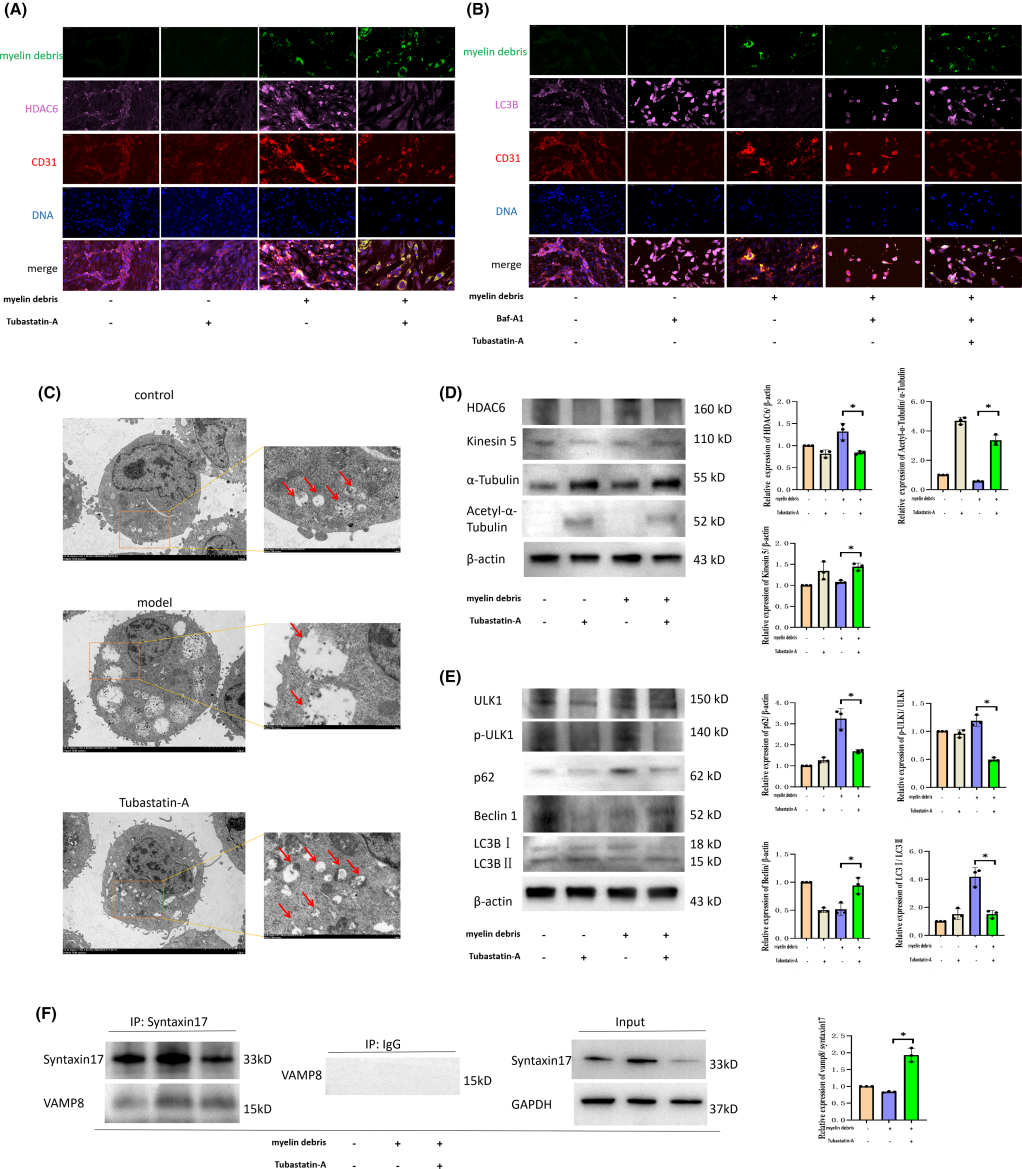
Tubastatin‐A promotes microvascular endothelial cells (MECs) to phagocytize myelin debris by regulating the HDAC6‐mediated autophagy‐lysosome pathway. (A) Tubastatin‐A was used to interfere with the coculture system. HDAC6 expression and the endocytosis of myelin debris were examined using immunofluorescence (myelin debris in green and HDAC6 in pink). (B) Tubastatin‐A and Baf‐A1 were used to interfere with the coculture system. LC3B expression and the endocytosis of myelin debris were examined using immunofluorescence (myelin debris in green and LC3B in pink). (C) The microstructure of MECs was observed using a transmission electron microscope. The red arrow indicates autophagy. (D, E) Expression levels of microtubule‐related and autophagy‐related proteins were examined via Western blotting. (F) The binding levels of Syntaxin17 and VAMP8 were examined using Co‐IP experiments.

Some studies have shown that the process by which MECs phagocytize myelin debris depends on the autophagy‐lysosome pathway^7^; however, the specific mechanisms underlying this process and the role of HDAC6 remain unclear. We first explored the relationship between HDAC6 expression and the efficiency with which MECs phagocytize myelin debris and found that HDAC6 expression was upregulated following the phagocytosis of myelin debris, although interference with Tubastatin‐A reduced this expression and promoted the endocytosis of myelin debris (Figure 5A). We then used Tubastatin‐A and Baf‐A1 (an autophagy inhibitor) to interfere with the co‐culture system, after which we examined the endocytosis of myelin debris and the expression of the structural autophagy protein LC3B via immunofluorescence multi‐labeling. Baf‐A1 treatment resulted in the accumulation of LC3B and reduced the endocytosis of myelin debris, while Tubastatin‐A attenuated this phenomenon (Figure 5B).

We also examined the autolysosome structure of MECs using transmission electron microscopy. Autolysosome swelling appeared after phagocytosis of myelin debris by MECs, which hindered the degradation process, while Tubastatin‐A attenuated this phenomenon (Figure 5C). To further explore the effect of HDAC6 on the intracellular degradation of MECs, we investigated the expression of microtubule system‐associated proteins (Kinesin5, α‐tubulin, acetyl‐α‐tubulin) and autophagy‐associated proteins (ULK1, p‐ULK1, p62, Beclin1, and LC3B) using western blotting. Tubastatin‐A increased the expression of Kinesin5 and acetyl‐α‐tubulin, reduced the accumulation of p62 and LC3B, and increased autophagic flux (Figure 5D,E). Finally, we performed Co‐IP experiments to examine the binding of Syntaxin17 and VAMP8. Tubastatin‐A improved the binding levels of Syntaxin17 and VAMP8 and promoted the fusion of autophagosomes and lysosomes (Figure 5F). These results indicate that inhibition of HDAC6 exerts regulatory effects on the autophagy‐lysosome pathway and promotes MECs to phagocytize myelin debris.

### 3.6 Phagocytosis of myelin debris by MECs in the early stages of SCI may affect nerve repair

**FIGURE 6:**
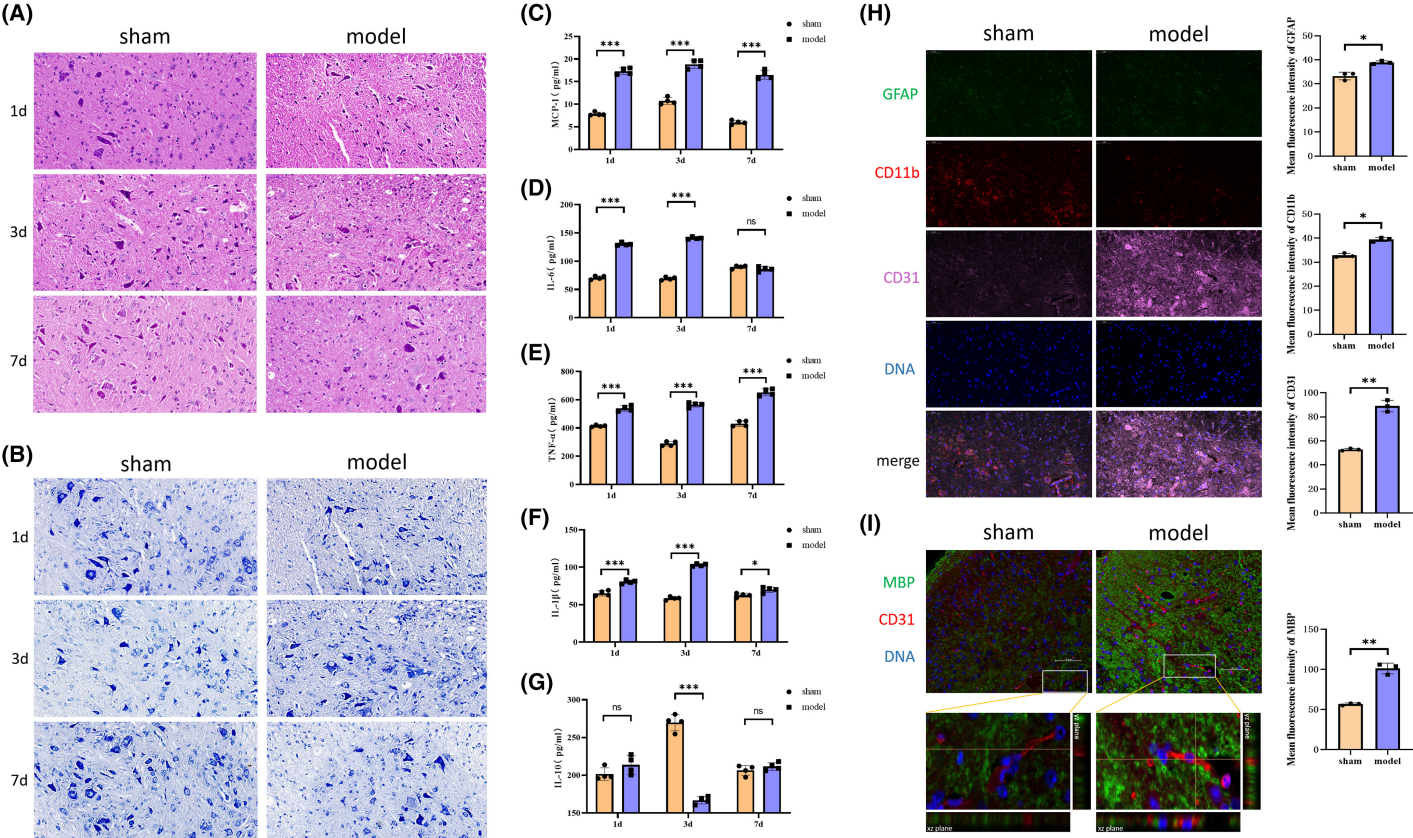
Phagocytosis of myelin debris by microvascular endothelial cells (MECs) may play a role in the repair of SCI. (A, B) Following the generation of the spinal cord injury (SCI) model, the injured spinal cord was removed for HE (overall structure and inflammatory infiltration) and Nissl staining (neuronal morphology and intracellular condition) on days 1, 3, and 7 postinjury. (C–G) On the first, third, and seventh days after SCI, levels of MCP‐1, IL‐6, IL‐1β, TNF‐α, and IL‐10 in the serum were examined via ELISA. (H) On the third day after SCI, immunofluorescence experiments were used to examine the distributions of microglia (CD11b, red), astrocytes (GFAP, green), and MECs (CD31, pink). (I) On the third day after SCI, phagocytosis of myelin debris by MECs was examined using 3D confocal microscopy. The process of myelin debris entering vascular‐like structures was observed using x‐y, y‐z, and x‐z views (MBP in green, CD31 in red). All data are expressed as the mean ± standard deviation (*n* ≥ 3 replicates per group). ns *p* > 0.05, *p < 0.05, **p < 0.01, ***p < 0.001.

To further explore the effects of the phagocytosis of myelin debris by MECs on SCI repair, we established an animal model of SCI. Hematoxylin and eosin (HE) staining and Nissl staining experiments were performed to investigate pathological changes in the injured spinal cord on days 1, 3, and 7 following the injury. In the model group, we observed a disordered arrangement of nerve fibers, infiltration of abundant inflammatory cells, pyknosis, and necrosis in some neurons. Consistent with previous findings, the most severe changes were observed on day 3 (Figure 6A,B).^1^ We then examined levels of pro‐inflammatory (MCP‐1, IL‐6, IL‐1β, and TNF‐α) and anti‐inflammatory factors (IL‐10) via ELISA. Our findings indicated that levels of pro‐inflammatory factors increased significantly following SCI, especially on the 3rd day, and that these changes were accompanied by significant decreases in anti‐inflammatory factors on day 3 (Figure 6C–G). Some authors have reported that large numbers of glial cells in the central area of SCI are lost due to mechanical injury,^2^, ^3^, ^4^, ^5^, ^23^ which may explain the failure to eliminate myelin debris. Therefore, we examined the local distribution of three types of cells in SCI via immunofluorescence, which revealed an absence of microglia and astrocytes on the third day after SCI, along with the proliferation of MECs (Figure 6H). This finding is in accordance with previously reported results.^2^ Furthermore, we assessed the phagocytosis of myelin debris by MECs in the local SCI region using 3D confocal microscopy, observing that MECs phagocytized myelin debris on day 3 after SCI (Figure 6I), which is also consistent with the findings of previous studies.^24^ Therefore, we speculate that phagocytosis of myelin debris by MECs in the early stages of SCI may affect the process of nerve repair.

### 3.7 Inhibition of HDAC6 regulates the immune‐inflammatory response and promotes MECs to phagocytize myelin debris

**FIGURE 7:**
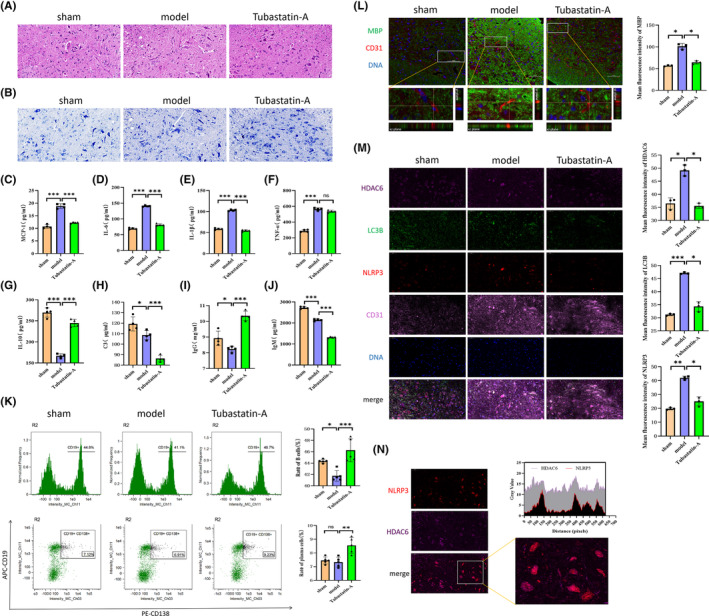
Tubastatin‐A promotes SCI repair by promoting microvascular endothelial cells (MECs) to phagocytize myelin debris and inhibit inflammatory secretion. (A, B) Tubastatin‐A was used to intervene in mice with spinal cord injury (SCI). On the third day after SCI, HE staining was performed to examine changes in global structure and inflammatory infiltration, while Nissl staining was performed to examine changes in neuronal morphology and intracellular conditions. (C–J) On the third day after SCI, ELISA was performed to examine changes in MCP‐1, IL‐6, IL‐1β, TNF‐α, IL‐10, C3, IgM, and IgG levels in the serum. (K) On the third day after SCI, flow cytometry was performed to examine the proliferation and differentiation of B cells in the mouse spleen. (L) On the third day after SCI, the phagocytosis of myelin debris by MECs was detected using 3D confocal microscopy. The process of myelin debris entering vascular‐like structures was observed using x‐y, y‐z, and x‐z views (MBP in green, CD31 in red). (M) On the third day after SCI, levels of HDAC6 (rose red), LC3B (green), NLRP3 (red), and CD31 (pink) expression were examined using immunofluorescence. (N) The co‐localization of HDAC6 (rose red) and NLRP3 (red) was detected based on the results of immunofluorescence experiments. All data are expressed as the mean ± standard deviation (*n* ≥ 3 replicates per group). ns *p* > 0.05, *p < 0.05, **p < 0.01, ***p < 0.001.

To explore precisely how HDAC6 influences SCI repair by regulating the immune‐inflammatory response, we performed interference experiments in SCI mice using Tubastatin‐A. On day 3 after SCI, HE and Nissl staining indicated that Tubastatin‐A reduced the infiltration of inflammatory cells as well as neuronal necrosis (Figure 7A,B). We then examined levels of pro‐inflammatory factors (MCP‐1, IL‐6, IL‐1β, TNF‐α, and C3), anti‐inflammatory factors (IL‐10), and immunoglobulins (IgG and IgM) via ELISA; Tubastatin‐A significantly reduced levels of pro‐inflammatory factors and increased levels of anti‐inflammatory factors. In addition, it significantly increased IgG levels while decreasing IgM levels (Figure 7C–J). These results were consistent with those of the previous cell experiments. We also observed that Tubastatin‐A promoted B cell proliferation and differentiation (Figure 7K), as well as the phagocytosis of myelin debris (Figure 7L). To further explore the mechanisms involved in this process, we examined the expression of the structural autophagy protein LC3B and the inflammasome marker NLRP3 using immunofluorescence. While levels of HDAC6, LC3B, and NLRP3 expression were upregulated in MECs after SCI, Tubastatin‐A inhibited the expression of these proteins (Figure 7M). Together, these results suggest that the anti‐inflammatory effect of Tubastatin‐A is related to the phagocytosis of myelin debris by MECs and the inhibition of the inflammasome pathway. In addition, there may be a direct interaction between NLRP3 and HDAC6 (Figure 7N), which is also consistent with previous findings.^17^

## 4 DISCUSSION

Secondary SCI results from ischemia, edema, inflammatory reactions, lipid peroxidation, apoptosis, and the influence of other factors highlight the importance of the inflammatory microenvironment in the pathological development of further injury.^25^ Inflammatory factors produced locally in the spinal cord have been shown to aggravate neuronal injury, inhibit nerve axon regeneration, and induce secondary injury after SCI.^26^ Therefore, reducing secondary injury by inhibiting inflammation in the acute stage of SCI may preserve residual nerve function and provide a favorable environment for nerve repair.

The myelin sheath sustains severe damage following SCI, producing a large amount of myelin debris within a short time while attracting certain molecules that can inhibit axon and myelin regeneration, thereby exerting a negative effect on nerve repair.^27^ Myelin debris acts as an inflammatory stimulant and aggravates secondary injury by activating microglia, macrophages, astrocytes, and other factors.^28^ Several studies have shown that accelerating the removal of myelin debris can improve the inflammatory microenvironment and may be key to promoting functional recovery after SCI.^29^, ^30^, ^31^, ^32^, ^33^ MECs are a recently identified cell type involved in the phagocytosis of myelin debris, which is opsonized by IgG and degraded by the intracellular autophagy‐lysosome pathway; however, myelin debris stimulates MECs to secrete inflammatory factors and hinders the repair process following SCI.^7^ Therefore, it is paramount for SCI research to devise strategies that promote MECs to phagocytize myelin debris while attenuating the secretion of inflammatory factors.

In the current study, we first constructed a coculture system of MECs and myelin debris to verify that phagocytosis of myelin debris by MECs triggers the secretion of inflammatory factors. Given the repulsive force between cells and foreign matter due to their negative charges, opsonized phagocytosis can promote the removal of foreign matter. Opsonin participates in the maintenance of inflammatory and anti‐inflammatory balance, with roles related to complement proteins (C3b, C4b), immunoglobulins (IgG, IgM), and C‐reactive protein.^34^ Some studies have shown that phagocytosis of myelin debris by MECs depends on the opsonization of IgG,^7^ while none have reported the involvement of IgM opsonization. Therefore, we conducted coculture experiments using IgM under various conditions, observing that myelin debris was also phagocytosed by MECs after opsonization by IgM, and that the efficiency of phagocytosis was proportional to IgM concentration. Notably, phagocytosis of myelin debris opsonized by IgM upregulated MCP‐1 levels, while that associated with IgG opsonization did not. These results suggest that MECs can phagocytize myelin debris opsonized by IgM, but this may aggravate the inflammatory response, highlighting the need for additional mechanistic studies.

Some studies have demonstrated that the expression of HDAC6 is upregulated following SCI, which impairs microtubule stability and leads to disruptions in autophagy‐lysosomal transport.^16^ Inhibition of HDAC6 attenuates these phenomena, downregulates the expression of IL‐1β and IL‐6, and reduces local inflammatory reactions.^17^, ^18^ Therefore, we used the HDAC6 inhibitor Tubastatin‐A to interfere with the coculture system and found that it promoted MECs to phagocytize myelin debris while reducing inflammation. We then used Tubastatin‐A to interfere with primary B cells and found that it inhibited the differentiation of B cells, increased the level of IgG, decreased the level of IgM, and downregulated the level of MCP‐1 secreted by MECs after phagocytosis of myelin debris. Given previous reports that B‐cell proliferation and differentiation mediated by the PI3K/Akt1/STAT3 axis may affect the inflammatory response,^35^ we suspected that the effects of Tubastatin‐A may be related to this axis. Thus, we explored the effect of Tubastatin‐A on the autophagy‐lysosome pathway. Tubastatin‐A promoted MECs to phagocytize myelin debris by regulating the autophagy‐lysosome pathway, mainly by increasing autophagy flux, fusion of autophagosomes and lysosomes, and endocytosis of myelin debris. Therefore, HDAC6 inhibition may represent a novel strategy for promoting MECs phagocytosis of myelin debris while simultaneously reducing the inflammatory response.

To further explore the effect of Tubastatin‐A on SCI repair, we established an SCI animal model. First, we observed significant increases in levels of pro‐inflammatory factors on day 3 after SCI, along with the accumulation of myelin debris at the lesion site. Although microglia and astrocytes were absent at this time, the proliferation of MECs was observed along with phagocytosis of myelin debris. Therefore, we speculated that the phagocytosis of myelin debris by MECs may affect the repair process during SCI's early stages. Furthermore, interference experiments in SCI mice revealed that Tubastatin‐A significantly reduced levels of pro‐inflammatory factors, reduced the accumulation of myelin debris, and improved the pathological structure of the spinal cord. Notably, Tubastatin‐A also promoted the proliferation and differentiation of B cells, significantly increased IgG levels, and reduced IgM levels. However, this is not completely consistent with the results of Tubastatin‐A intervention in the cellular experiment, possibly because in vitro experiments cannot simulate the complexity of the in vivo environment. Proper control of the immune response following SCI is generally considered to promote repair, although the consequences of the immune response remain controversial, with some reports suggesting benefits^36^, ^37^, ^38^ and others suggesting detriments.^39^, ^40^, ^41^, ^42^ Although our results provide relevant support for the beneficial aspects of the immune response, the specific mechanisms underlying these effects require further exploration. Finally, we observed that the anti‐inflammatory effects of Tubastatin‐A were related to its ability to promote the phagocytosis of myelin debris by MECs and inhibit the NLRP3 inflammasome pathway. Some studies have suggested that inhibition of HDAC6 attenuates neuroinflammation,^18^ whereas others have reported direct binding between HDAC6 and NLRP3.^17^ Whether this process occurs during the phagocytosis of myelin debris by MECs remains to be determined.

In conclusion, the current results indicate that inhibition of HDAC6 after SCI regulates B cell proliferation and differentiation, upregulates IgG, downregulates IgM, and reduces inflammatory factors secreted by MECs following the phagocytosis of myelin debris. Conversely, inhibition of HDAC6 regulates the autophagy‐lysosome pathway to promote MECs to phagocytize myelin debris and reduce the secretion of inflammatory factors. Based on these two findings, inhibition of HDAC6 to regulate the immune‐inflammatory response and promote MECs to phagocytize myelin debris may represent a novel strategy in the treatment of SCI.

## AUTHOR CONTRIBUTIONS

CW and YP conceptualized the study's experiments and wrote the manuscript. PT, SC, LS, and DY provided information for the research design. LW and ML contributed to the data analysis. YG and YM designed this study and edited the final manuscript. All authors contributed to the article and approved the submission.

## FUNDING INFORMATION

This work was supported by grants from the National Natural Science Foundation of China (81973885), Natural Science Foundation Youth Project of Nanjing University of Chinese Medicine (NZY81704100), Jiangsu Province University Natural Science Foundation Project (21KJB360006), 2022 Jiangsu Province Graduate Research and Practice Innovation Project (KYCX22_1942), Traditional Chinese and Western Medicine Clinical Medicine Brand Construction Project of Jiangsu Higher Education Institutions (Phase II) (2020PPZXL261).

## CONFLICT OF INTEREST STATEMENT

The authors declare that the research was conducted in the absence of any commercial or financial relationships that could be construed as a potential conflict of interest.

## Supporting information


Data S1:

## Data Availability

All relevant data are available and can be provided upon request to the corresponding authors.
