# Supplementary material for: Inhibition of HDAC6 promotes microvascular endothelial cells to phagocytize myelin debris and reduces inflammatory response to accelerate the repair of spinal cord injury
Source: CNS Neurosci Ther. 2023 Aug 29;30(3):e14439. doi: 10.1111/cns.14439 (PMC10916453; doi:10.1111/cns.14439)

Full unedited gel/blot for Figure 3 G

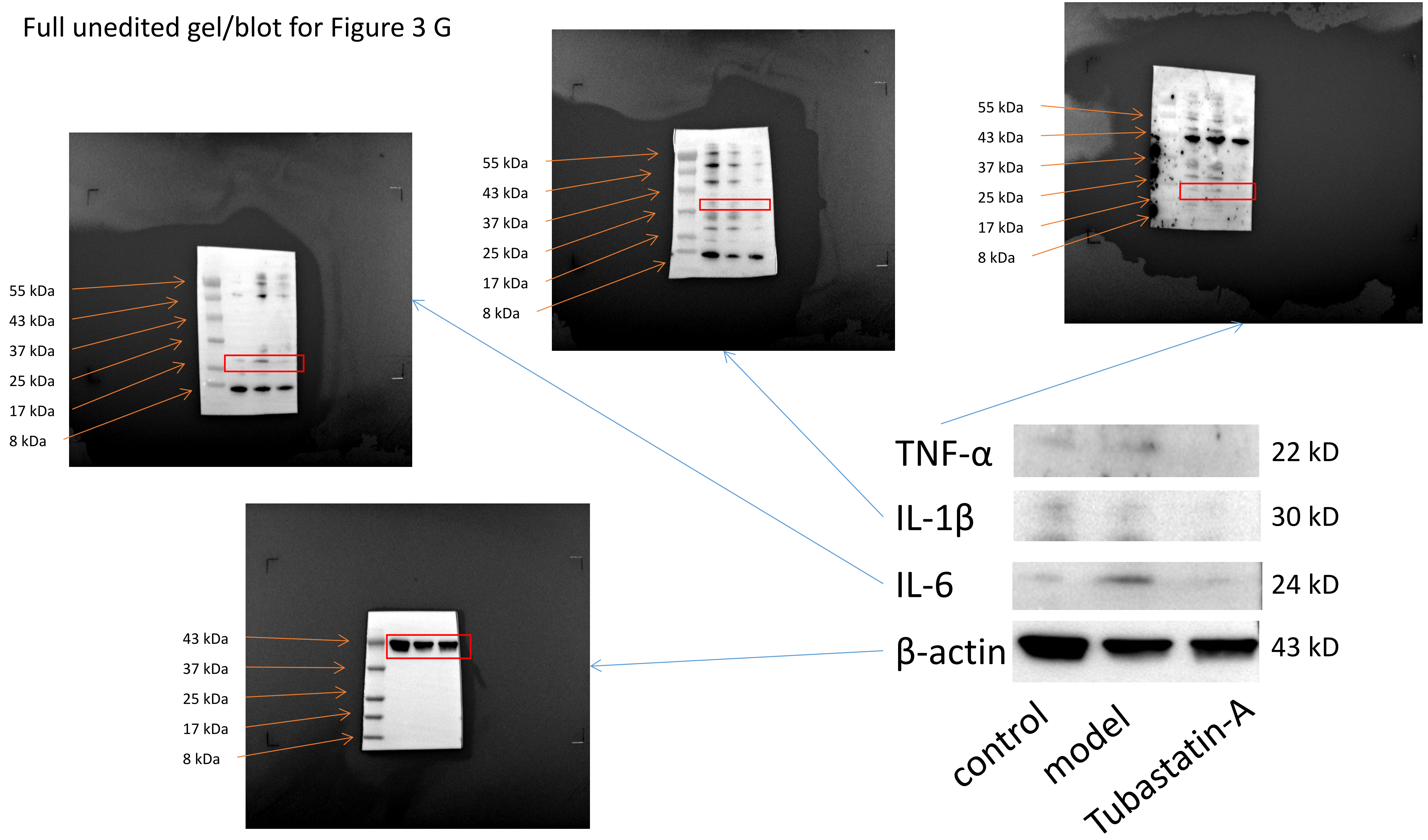

Full unedited gel/blot for Figure 5 D

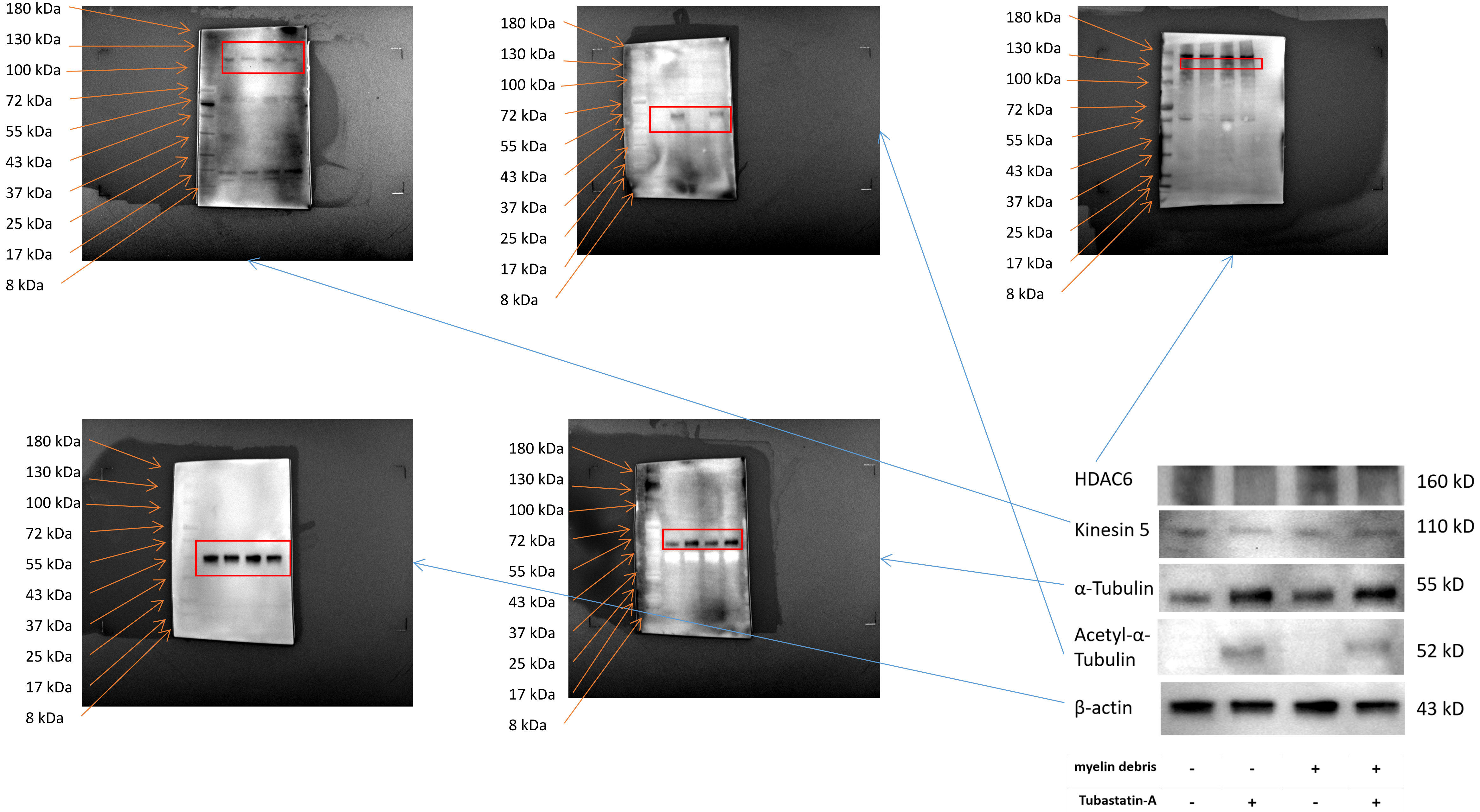

Full unedited gel/blot for Figure 5 E

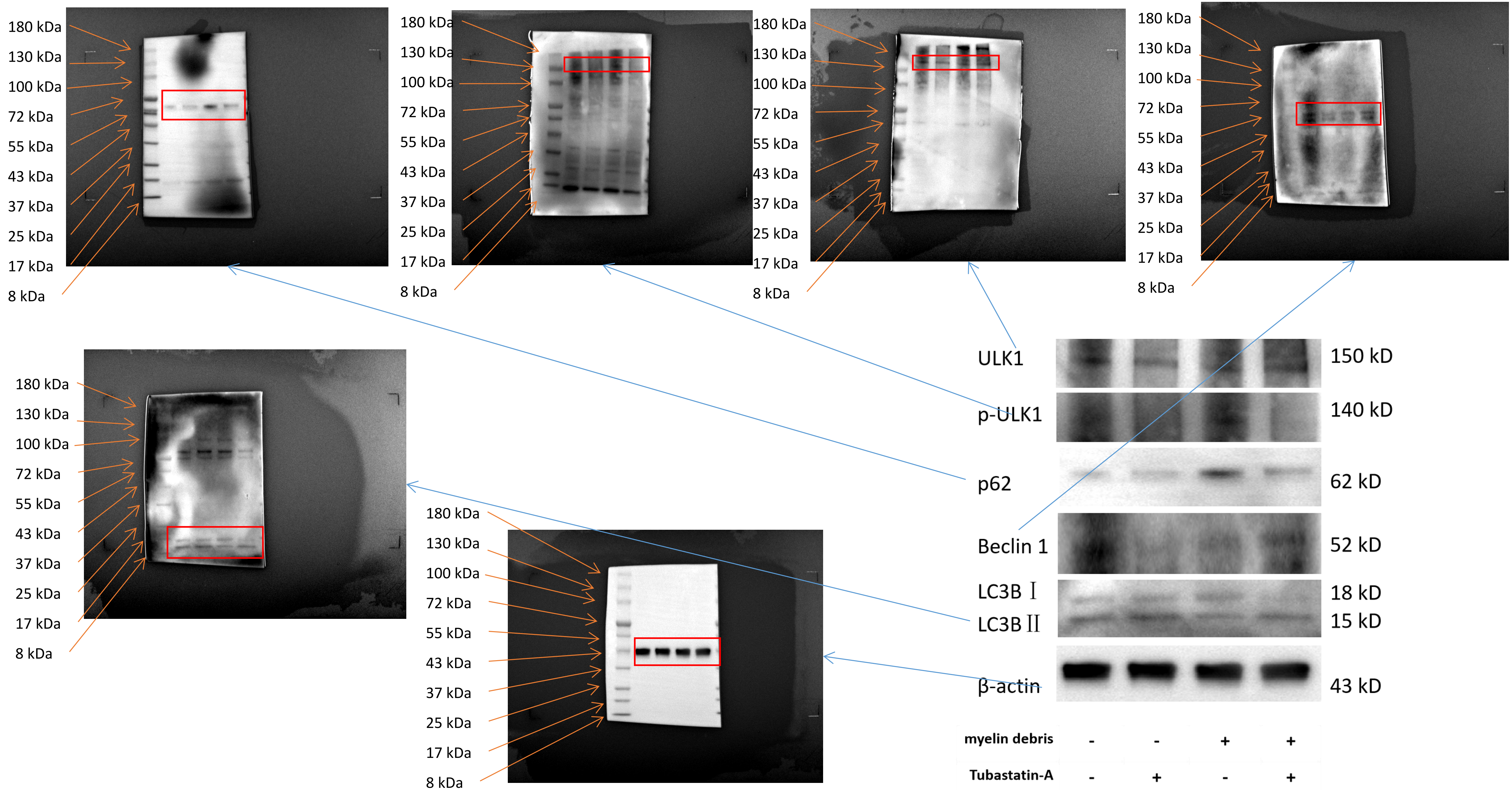

Full unedited gel/blot for Figure 5 F

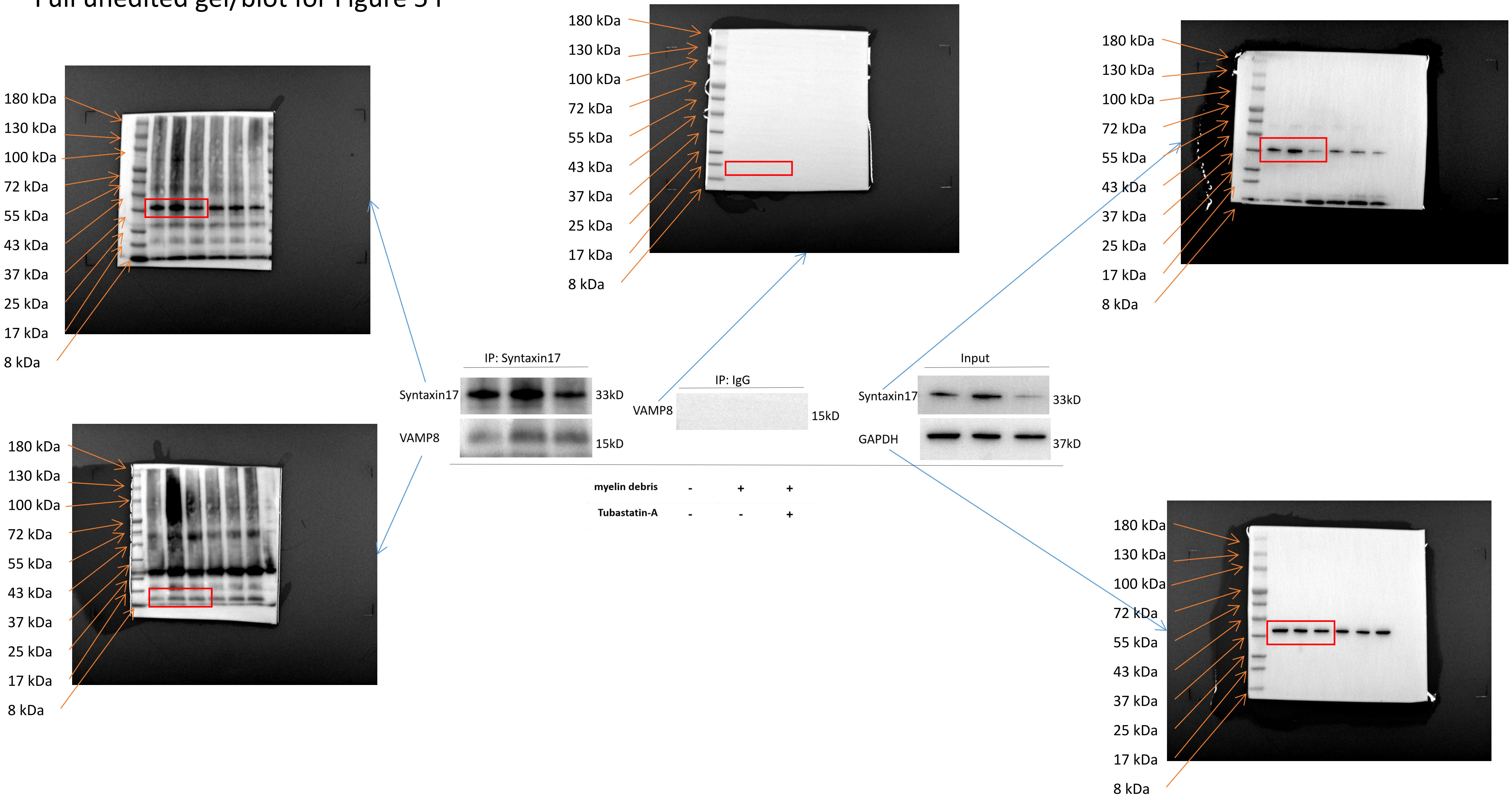

Supplement: Supplementary file 1 — Data S1: [file CNS-30-e14439-s001.pdf]
